# Molecular Mechanisms for Microbe Recognition and Defense by the Red Seaweed *Laurencia dendroidea*

**DOI:** 10.1128/mSphere.00094-17

**Published:** 2017-12-06

**Authors:** Louisi Souza de Oliveira, Diogo Antonio Tschoeke, Ana Carolina Rubem Magalhães Lopes, Daniela Bueno Sudatti, Pedro Milet Meirelles, Cristiane C. Thompson, Renato Crespo Pereira, Fabiano L. Thompson

**Affiliations:** a Instituto de Biologia, Universidade Federal do Rio de Janeiro (UFRJ), Rio de Janeiro, Brazil; b Núcleo em Ecologia e Desenvolvimento Sócio-Ambiental de Macaé (NUPEM), Universidade Federal do Rio de Janeiro, Macaé, Rio de Janeiro, Brazil; c Departamento de Biologia Marinha, Universidade Federal Fluminense (UFF), Niterói, Rio de Janeiro, Brazil; Aix-Marseille University

**Keywords:** bacteria, cell signaling, defense, differential expression, seaweed, terpenes

## Abstract

The ability to recognize and respond to the presence of microbes is an essential strategy for seaweeds to survive in the marine environment, but understanding of molecular seaweed-microbe interactions is limited. *Laurencia dendroidea* clones were inoculated with the marine bacterium *Vibrio madracius*. The seaweed RNA was sequenced, providing an unprecedentedly high coverage of the transcriptome of *Laurencia*, and the gene expression levels were compared between control and inoculated samples after 24, 48, and 72 h. Transcriptomic changes in *L. dendroidea* in the presence of *V. madracius* include the upregulation of genes that participate in signaling pathways described here for the first time as a response of seaweeds to microbes. Genes coding for defense-related transcription activators, reactive oxygen species metabolism, terpene biosynthesis, and energy conversion pathways were upregulated in inoculated samples of *L. dendroidea*, indicating an integrated defensive system in seaweeds. This report contributes significantly to the current knowledge about the molecular mechanisms involved in the highly dynamic seaweed-bacterium interactions.

**IMPORTANCE** Marine bacteria are part of the healthy microbiota associated with seaweeds, but some species, such as *Vibrio* spp., are frequently associated with disease outbreaks, especially in economically valuable cultures. In this context, the ability of seaweeds to recognize microbes and, when necessary, activate defense mechanisms is essential for their survival. However, studies dedicated to understanding the molecular components of the immune response in seaweeds are rare and restricted to indirect stimulus. This work provides an unprecedentedly large-scale evaluation of the transcriptional changes involved in microbe recognition, cellular signaling, and defense in the red seaweed *Laurencia dendroidea* in response to the marine bacterium *Vibrio madracius*. By expanding knowledge about seaweed-bacterium interactions and about the integrated defensive system in seaweeds, this work offers the basis for the development of tools to increase the resistance of cultured seaweeds to bacterial infections.

## Abstract (precis)

Marine bacteria are part of the healthy microbiota associated with seaweeds, but some species, such as *Vibrio* spp., are frequently associated with disease outbreaks, especially in economically valuable cultures. In this context, the ability of seaweeds to recognize microbes and, when necessary, activate defense mechanisms is essential for their survival. However, studies dedicated to understanding the molecular components of the immune response in seaweeds are rare and restricted to indirect stimulus. This work provides an unprecedentedly large-scale evaluation of the transcriptional changes involved in microbe recognition, cellular signaling, and defense in the red seaweed *Laurencia dendroidea* in response to the marine bacterium *Vibrio madracius*. By expanding knowledge about seaweed-bacterium interactions and about the integrated defensive system in seaweeds, this work offers the basis for the development of tools to increase the resistance of cultured seaweeds to bacterial infections.

## INTRODUCTION

Seaweeds are extremely susceptible to microbial colonization due to the release of large amounts of carbon compounds that act as chemical attractants and nutrient sources for bacteria (1). The microbial community associated with seaweeds tends to be species specific and different from that associated with seawater (2). The microbiome associated with healthy individuals of the red seaweed *Laurencia dendroidea* can fix nitrogen and provide relevant amino acids and vitamins to the seaweed (3). The tight association between seaweeds and their epiphytic microbes led to the establishment of a holobiont concept that is analogous to that corresponding to the well-described microbe-coral relationship (4). However, potential pathogens were also previously detected on seaweed thalli and include microorganisms capable of degrading cell wall polysaccharides (5–7). Diseases can significantly impact host populations by promoting a decrease of individual fitness and negatively affecting the ability of seaweeds to defend against herbivores (8). Besides, the occurrence of disease outbreaks in valuable reared seaweeds, such as *Porphyra* (nori) cultures, causes significant economic losses due to a reduction of annual production (9).

Seaweed’s defense against microbes involves a multilevel strategy that starts with the recognition of microbe-associated molecular patterns (MAMPs) or pathogen-induced molecular patterns (PIMPs). Overall, MAMPs include conserved molecules that are characteristic of microbes but are absent in hosts, e.g., bacterial cell wall components (peptidoglycans, lipoteichoic acid, and lipopolysaccharides) or flagellin (10), while PIMPs are the products of the microbial degradation of seaweed cell wall matrix, including oligoagars and oligoguluronates (11). Following the recognition of microbes, evidence has emerged for the occurrence and significant role of innate immunity processes as the first line of defense in seaweeds, similarly to that observed in vascular plants and metazoans (10–13), including transient production of reactive oxygen species (ROS) (14–16). Besides being directly toxic to microbes (17), ROS participate in intracellular signaling mechanisms leading to the activation of other defense responses (18), such as the expression of genes related to the biosynthesis of secondary metabolites (19). Despite being part of the defensive strategy of seaweeds against fouling (20), the presence of ROS can damage the seaweed cell structures, so the oxidative burst must be tightly regulated through the activation of antioxidant enzymes (21).

Molecular studies in seaweeds have had mixed results regarding the potential costs involved in defense. For example, an increase in the expression of genes involved in cellular energy was detected through suppression subtractive hybridization (SSH) following the exposure of *Laminaria digitata* to oligoguluronates (19). In contrast, the downregulation of genes involved in energy conversion was detected, through a microarray, after the exposure of *Chondrus crispus* to methyl jasmonate (22). The conflicting results could be attributed to intrinsic biological differences between the two species or to the relatively small number of sequences analyzed.

*Laurencia* is a red seaweed genus widely distributed around the world, recognized for the biosynthesis of diverse halogenated secondary metabolites, especially terpenes, with relevant ecological (23, 24) and pharmacological (25–29) activities. Some of these halogenated compounds are able to prevent the growth of marine bacteria (30–32). Vairappan et al. (32) reported the dominance of *L. majuscula* during a disease outbreak; its dominance was attributed to the synthesis of secondary metabolites with antibiotic effects. Accordingly, disease symptoms were not observed in natural populations of *L. dendroidea*. The halogenated metabolites in *L. dendroidea* are stored inside vacuolar cell structures called *corps en cerise* (CC) (33), and they are released to the cell surface through regulated vesicle trafficking (34), which can be induced by microbes (35). The compartmentation of secondary metabolites in vacuoles, possibly to avoid autotoxicity, was previously observed in plants and other seaweeds (36, 37). However, the genes involved in this cellular process are still largely unknown. Although a large array of genes responsible for the biosynthesis of terpenes was recently characterized in *L. endroidea* (38), the molecular mechanisms involved in the response of *Laurencia* species to bacteria are still largely unknown.

*Vibrio* is a genus of Gram-negative bacteria associated with ice-ice disease in several red seaweeds, such as *Kappaphycus alvarezii* and *Eucheuma denticulatum* (5), and also with hole-rotten disease in the brown seaweed *Laminaria japonica* (39). *Vibrio madracius* is phylogenetically close to the *V. mediterranei* species (40), previously reported to cause bleaching in corals (41–43). Additionally, *V. madracius* is associated with bleached coral (*Madracis decactis*) (40), indicating that this bacterial species would have a deleterious effect on the symbiotic algae. Oxidative stress resistance proteins are necessary in the pathogenic marine *Vibrio* species for the progression of virulence (44). Because *V. madracius* is oxidase and catalase positive, it may tolerate ROS defense responses and colonize algae.

Current knowledge about seaweed-microbe interactions at the molecular level is limited, because studies evaluating seaweed resistance to pathogens have been based on the use of indirect stimulus through the application of MAMPs (16), PIMPS (15, 19, 45, 46), and signaling molecules (e.g., arachidonic acid, linolenic acid, and methyl jasmonate) (22, 47, 48). Recently, an initial attempt to understand the global effects of microbes on a seaweed transcriptome was indirectly made using an agarolytic enzyme (49). Nonetheless, the direct effects of microorganisms on seaweed gene expression have rarely been evaluated and have relied on real-time PCR techniques, monitoring a limited number of genes (49, 50). The dynamic nature of seaweed’s molecular response to microbes implicates temporal complexity and metabolic shifts. Our aim was to identify the major transcriptional responses of *L. endroidea* in the presence of *V. madracius*.

## RESULTS

The transcriptome sequencing of *L. dendroidea* 24 h, 48 h, and 72 h after *V. madracius* inoculation in the culture medium resulted in 12.58 Gbp, which represents approximately 190-fold coverage of the transcriptome of *L. endroidea*, considering a genome size estimate of 833 Mbp (51) and that 8% of the genetic material codes for proteins (as described for *Chondrus crispus* by Collén et al. [52]). After the preprocessing step, the sequences were *de novo* assembled, resulting in 151,740 sequences that were grouped into 53,677 clusters, which are referred to here as genes (Table 1). A total of 36.28% of the genes were shared among all of the control samples regardless of the time that had elapsed since the beginning of the experiment, while 3.79% of the genes were shared among all of the inoculated samples (see Fig. S1 in the supplemental material). We detected in both the control (uninoculated) samples and the samples of *L. dendroidea* inoculated with *V. madracius* the expression of genes coding for leucine-rich repeat receptor-like serine/threonine-protein kinase (LRR-RLK) (Fig. S2).

10.1128/mSphere.00094-17.1 FIG S1  Numbers of genes shared among control samples (left, 7 samples) and samples of *Laurencia dendroidea* inoculated with *Vibrio madracius* (right, 8 samples). Download FIG S1, EPS file, 1.5 MB. Copyright © 2017 de Oliveira et al. 2017 de Oliveira et al. This content is distributed under the terms of the Creative Commons Attribution 4.0 International license.

10.1128/mSphere.00094-17.2 FIG S2  Conserved domains detected for LRR receptor-like serine/threonine-protein kinase candidate from *Laurencia dendroidea*. PLN00113, leucine-rich repeat receptor-like protein kinase provisional (E value, 1.70 e−29); LRR, leucine-rich repeat (LRR) protein (transcription) (2.49 e−06); LRR_8, leucine rich repeat (7.14 e−04). Download FIG S2, TIF file, 0.8 MB. Copyright © 2017 de Oliveira et al. 2017 de Oliveira et al. This content is distributed under the terms of the Creative Commons Attribution 4.0 International license.

**TABLE 1:** Characteristics of the cDNA sequences from *Laurencia dendroidea* after preprocessing and assembly^a^

Parameter	Value(s)						
	Ctrl. 24 h (*n* = 2)	InOC. 24 h (*n* = 3)	Ctrl. 48 h (*n* = 2)	InOC. 48 h (*n* = 2)	Ctrl. 72 h (*n* = 3)	InOC. 72 h (*n* = 3)	Assembled sequences
Total nucleotides (Mbp)	1,981	3,856	2,606	2,492	2,177	2,328	91.46
No. of sequences	6,016,980	12,040,124	8,266,332	7,941,106	12,588,310	13,655,346	151,740
Avg sequence size (bp) ± SD	172.5 ± 63.4	168.8 ± 59.1	164.1 ± 59.5	163.2 ± 58.3	172.6 ± 49.4	170.1 ± 49.9	602 ± 674

a Ctrl., uninoculated samples; InOC., inoculated samples 24 h, 48 h, and 72 h after inoculation with *V. madracius*; SD, standard deviation.

**FIG 1:**
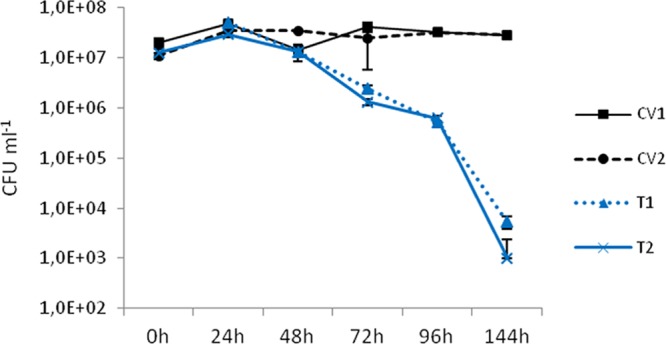
Concentration of *Vibrio madracius* in the culture medium in the presence (2 replicates [T1 and T2]) and absence (2 replicates [CV1 and CV2]) of *Laurencia dendroidea*. The concentration of *V. madracius* is presented as the number of colony-forming units per milliliter of culture medium as measured for 144 h after bacterial inoculation (average ± standard error).

The number of differentially expressed genes in the seaweed *L. endroidea* was maximal 24 h after *V. madracius* inoculation, and the transcriptomic profile tended to be similar to that seen with the control condition 72 h postinoculation (hpi). The concentration of *V. madracius* in the culture medium was reduced progressively after 72 h in the presence of *L. dendroidea* (Fig. 1). Plating the seaweed tissue homogenate on thiosulfate-citrate-bile salts-sucrose (TCBS) media did not result in bacterial growth, suggesting that this reduction was not due to bacterial attachment to *L. dendroidea* thalli.

**FIG 2:**
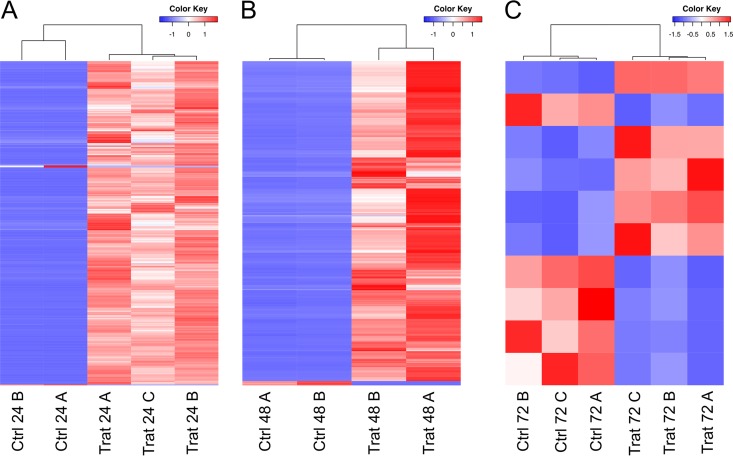
Heat map of expression values (*Z* score) for differentially expressed genes in *Laurencia dendroidea* 24 h (A), 48 h (B), and 72 h (C) after *Vibrio madracius* inoculation. Both annotated and nonannotated genes are represented. The analysis was based on the following numbers of replicates: control 24 h = 2, inoculated 24 h = 3, control 48 h = 2, inoculated 48 h = 2, control 72 h = 3, inoculated 72 h = 3.

The comparative analysis of control and inoculated specimens of *L. dendroidea* revealed the change in the gene expression profile in response to *V. madracius*. Most of the genes differentially expressed were upregulated in the inoculated samples of *L. dendroidea*, especially 24 and 48 hpi, while we verified a significant reduction in the number of genes differentially expressed in *L. dendroidea* 72 h after *V. madracius* inoculation. Overall, at 24 hpi, we observed in *L. dendroidea* the upregulation of 675 genes, of which 75.8% were annotated and 6 (16.7% annotated) were downregulated (Fig. 2A). In addition, 48 h after *V. madracius* inoculation, 299 genes were upregulated, of which 82.3% were annotated and 4 (annotated as encoding hypothetical proteins) were downregulated (Fig. 2B). Finally, 72 h after the introduction of *V. madracius* in the culture medium, the expression level of 5 genes increased, but none of them were identified through Blast, and 5 genes were repressed, of which 60% were annotated at the protein family level at least (Fig. 2C).

**FIG 3:**
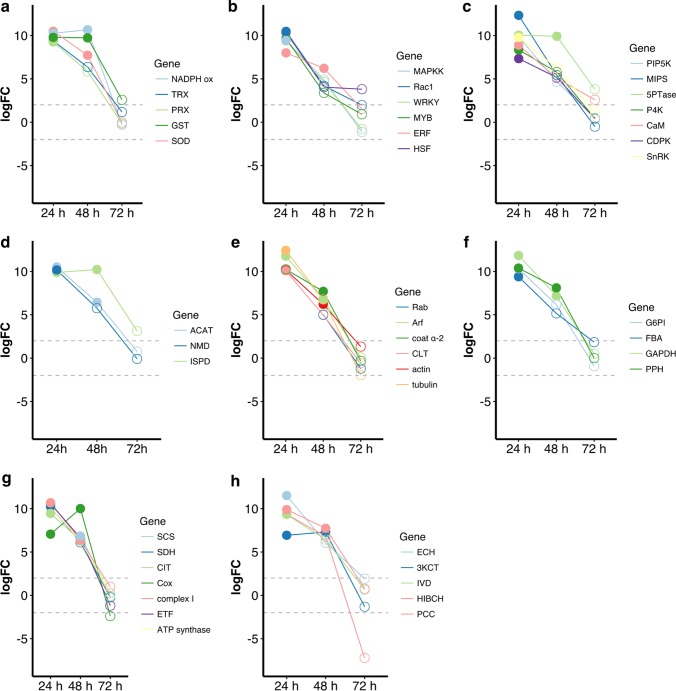
Relevant differentially expressed genes in *Laurencia dendroidea* 24, 48, and 72 h after inoculation with *Vibrio madracius* (data represent logFC values considering “inoculated” versus “control” samples). (a) Products of genes involved in oxidative burst and antioxidant mechanisms are indicated as follows: NADPH oxidase, NADPH ox; thioredoxin, TRX; peroxiredoxin, PRX; glutathione *S*-transferase, GST; superoxide dismutase, SOD. (b) Products of genes involved in the MAPK cascade and small GTPase-mediated signaling and transcription factors are indicated as follows: mitogen-activated protein kinase kinase, MAPKK; Rho-related protein, Rac1; transcription factor WRKY; transcription factor MYB; ethylene-responsive transcription factor, ERF; heat stress transcription factor, HSF. (c) Products of genes related to phosphoinositide and calcium-dependent signaling are indicated as follows: phosphatidylinositol 4-phosphate 5-kinase, PIP5K; myo-inositol 1-phosphate synthase, MIPS; type II inositol 1,4,5-trisphosphate 5-phosphatase, 5PTase; phosphatidylinositol 4-kinase, P4K; calmodulin, CaM; calcium calmodulin-dependent protein kinase, CDPK; Snf1-related protein kinase, SnRK. (d) Products of genes that participate in the biosynthesis of terpenes are indicated as follows: acetyl-CoA C-acetyltransferase, ACAT; (+)-neomenthol dehydrogenase, NMD; (-)-isopiperitenol dehydrogenase, ISPD. (e) Products of genes involved in vesicle trafficking are indicated as follows: Rab GTPase, Rab; ADP-ribosylation factor, Arf; coatomer, coat α-2; clathrin, CLT; actin, tubulin. (f) Products of genes involved in glycolysis are indicated as follows: glucose-6-phosphate isomerase, G6PI; fructose-bisphosphate aldolase, FBA; glyceraldehyde-3-phosphate dehydrogenase, GAPDH; phosphopyruvate hydratase, PPH. (g) Products of genes involved in tricarboxylic acid cycle and oxidative phosphorylation are indicated as follows: succinyl-CoA ligase, SCS; succinate dehydrogenase, SDH; citrate synthase, CIT; cytochrome *c* oxidase, Cox; NADH-ubiquinone oxidoreductase, complex I; electron transfer flavoprotein, ETF; ATP synthase. (h) Products of genes related to fatty acid oxidation and branched-chain amino acid catabolism are indicated as follows: enoyl-CoA hydratase, ECH; 3-ketoacyl-CoA thiolase, 3KCT; isovaleryl-CoA dehydrogenase, IVD; 3-hydroxyisobutyryl-CoA hydrolase, HIBCH; propionyl-CoA carboxylase, PCC. Open circles indicate values of logFC that were not statistically significant (*P* value = >0.001; logFC = <|2.0|). Numbers of replicates were as follows: control 24 h = 2, inoculated 24 h = 3, control 48 h = 2, inoculated 48 h = 2, control 72 h = 3, inoculated 72 h = 3.

The gene coding for NADPH oxidase (NADPH ox), which is responsible for transient production of ROS, was upregulated in *L. dendroidea* 24 hpi (Fig. 3a). At 24 and 48 hpi, we also observed the upregulation of the genes coding for antioxidant enzymes, such as thioredoxin (TRX), peroxiredoxin (PRX), glutathione *S*-transferase (GST), and superoxide dismutase (SOD) (Fig. 3a), and of genes associated with protein folding (Fig. S4a).

The gene coding for a mitogen-activated protein kinase kinase (MAPKK) was upregulated 24 hpi (Fig. 3b). Another relevant biological process overrepresented at 24 and 48 h after *V. madracius* inoculation was “small GTPase-mediated signal transduction” (Fig. S3), which included Rho-related protein rac1 (Fig. 3b). Additionally, genes coding for phosphatidylinositol 4-phosphate 5-kinase (PIP5K), myo-inositol 1-phosphate synthase (MIPS), type II inositol 1,4,5-trisphosphate 5-phosphatase (5PTase), phosphatidylinositol 4-kinase (P4K), calmodulin (CaM), calcium calmodulin-dependent protein kinase (CDPK), and Snf1-related protein kinase (SnRK) were upregulated mainly 24 hpi (Fig. 3c). The genes coding for WRKY, MYB, ethylene-responsive transcription factor (ERF), and heat stress transcription factor (HSF) were upregulated 24 h and 48 hpi (Fig. 3b).

10.1128/mSphere.00094-17.3 FIG S3  Overrepresented biological processes in *Laurencia dendroidea* 24 and 48 h after *Vibrio madracius* inoculation. Numbers of replicates were as follows: control 24 h = 2, inoculated 24 h = 3, control 48 h = 2, inoculated 48 h = 2. Download FIG S3, JPG file, 1 MB. Copyright © 2017 de Oliveira et al. 2017 de Oliveira et al. This content is distributed under the terms of the Creative Commons Attribution 4.0 International license.

Several genes related to the biosynthesis of terpenes were also upregulated in *L. dendroidea* in response to *V. madracius*, such as the genes coding for acetyl-CoA C-acetyltransferase (ACAT) 24 and 48 h after inoculation and genes homologous to those coding for plant (-)-isopiperitenol dehydrogenase (ISPD) 24 hpi and (+)-neomenthol dehydrogenase (NMD) 24 and 48 hpi (Fig. 3d). Twelve genes involved in the biosynthesis of terpenoid backbones and 10 genes involved in the biosynthesis of monoterpenes (C10), sesquiterpenes (C15), diterpenes (C20), and triterpenes (C30) were detected for the first time in *Laurencia* (Table 2). Genes coding for the Ras-related protein Rab, ADP-ribosylation factor (Arf), coatomer (coat α-2), and clathrin (CLT) were distributed in the categories “transport” and “intracellular protein transport” and upregulated in the seaweed in response to *V. madracius* (Fig. 3e, Fig. S3).

**TABLE 2:** Genes related to the biosynthesis of terpenes characterized for the first time in *Laurencia dendroidea* with their EC number, Blast E value, identity, and similarity and the metabolic pathway in which they participate

Gene product	EC no.	Blast e-value	% identity	% similarity	Biosynthetic pathway
Hydroxymethylglutaryl-CoA synthase	2.3.3.10	2.00 e−48	36	56	Terpenoid backbone
Hydroxymethylglutaryl-CoA reductase	1.1.1.34/1.1.1.88	3.00 e−61	73	87	Terpenoid backbone
Phosphomevalonate kinase	2.7.4.2	1.00 e−59	32	44	Terpenoid backbone
Diphosphomevalonate decarboxylase	4.1.1.33	3.00 e−99	55	69	Terpenoid backbone
Isopentenyl phosphate kinase	2.7.4.26	3.00 e−20	26	49	Terpenoid backbone
(2Z,6E)-farnesyl diphosphate synthase	2.5.1.68	3.00 e−80	44	63	Terpenoid backbone
(2E,6E)-farnesyl diphosphate synthase	2.5.1.10	1.00 e−95	47	65	Terpenoid backbone
Prenylcysteine oxidase	1.8.3.5	6.00 e−93	37	56	Terpenoid backbone
Hexaprenyl diphosphate synthase (geranylgeranyl-diphosphate specific)	2.5.1.82	2.00 e−60	43	59	Terpenoid backbone
Heptaprenyl diphosphate synthase	2.5.1.30	2.00 e−14	43	63	Terpenoid backbone
Undecaprenyl diphosphate synthetase	2.5.1.31	1.00 e−43	49	64	Terpenoid backbone
All-*trans*-octaprenyl-diphosphate synthase	2.5.1.90	2.00 e−28	38	59	Terpenoid backbone
Linalool 8-monooxygenase	1.14.13.151	4.00 e−13	35	58	Monoterpenoid
(-)-Isopiperitenol dehydrogenase	1.1.1.223	9.00 e−24	33	50	Monoterpenoid
(+)-Menthofuran synthase	1.14.13.104	5.00 e−19	30	47	Monoterpenoid
(+)-Neomenthol dehydrogenase	1.1.1.208	4.00 e−23	31	49	Monoterpenoid
Germacrene a hydroxylase	1.14.13.123	1.00 e−29	28	50	Sesquiterpenoid
Ent-cassa-12,15-diene 11-hydroxylase	1.14.13.145	5.00 e−10	38	57	Diterpenoid
Ent-kaurene oxidase	1.14.13.78	4.00 e−22	40	53	Diterpenoid
Ent-kaurenoic acid oxidase	1.14.13.79	3.00E-6	38	59	Diterpenoid
Squalene monooxygenase	1.14.14.17	8.00 e−85	50	67	Triterpenoid
11-Oxo-beta-amyrin 30-oxidase	1.14.13.173	6.00 e−38	27	43	Triterpenoid

Functional categories associated with energy conversion, such as the glycolytic process, including glucose-6-phosphate isomerase (G6PI), fructose-bisphosphate aldolase (FBA), glyceraldehyde-3-phosphate dehydrogenase (GAPDH), and phosphopyruvate hydratase (PPH), were overrepresented in the transcriptome of *L. dendroidea* 24 h and 48 h after the introduction of *V. madracius* in the culture medium (Fig. 3f). Further, genes related to the tricarboxylic acid cycle and oxidative phosphorylation, e.g., those coding for succinyl-CoA ligase (SCS), succinate dehydrogenase (SDH), citrate synthase (CIT), cytochrome *c* oxidase (Cox), NADH-ubiquinone oxidoreductase (complex I), electron transfer flavoprotein (ETF), and ATP synthase, were upregulated in *L. dendroidea* 24 and 48 hpi (Fig. 3g, Fig. S4b and c). Finally, at 24 and 48 hpi, we detected the upregulation of genes related to fatty acid oxidation and the catabolism of leucine, isoleucine, and valine, such as those coding for enoyl-CoA hydratase (ECH), 3-ketoacyl-CoA thiolase (3KCT), isovaleryl-CoA dehydrogenase (IVD), 3-hydroxyisobutyryl-CoA hydrolase (HIBCH), and propionyl-CoA carboxylase (PCC) (Fig. 3h, Fig. S4d).

10.1128/mSphere.00094-17.4 FIG S4  Genes differentially expressed in *Laurencia dendroidea* 24, 48, and 72 h after inoculation with *Vibrio madracius*. Numbers of replicates were as follows: control 24 h = 2, inoculated 24 h = 3, control 48 h = 2, inoculated 48 h = 2, control 72 h = 3, inoculated 72 h = 3. Download FIG S4, EPS file, 1.5 MB. Copyright © 2017 de Oliveira et al. 2017 de Oliveira et al. This content is distributed under the terms of the Creative Commons Attribution 4.0 International license.

## DISCUSSION

### Microbial recognition and ROS production.

The concentration of *V. madracius* reduced progressively after 72 h in the presence of *L. dendroidea*. Because this reduction could not be attributed to biofilm formation, we hypothesized that it should have been due to responses of or defense strategies activated in *L. dendroidea* in the first 72 h after *V. madracius* inoculation, as a consequence of recognizing the bacteria through specific membrane receptors. Pattern recognition receptors are largely unknown in seaweeds. A recent study demonstrated the occurrence of genes coding for LRR kinases in the brown seaweed *Ectocarpus siliculosus* that, due to their molecular structure, were considered to represent candidate pathogen receptors (53). Here, we detected, in both control and inoculated samples, the expression of genes coding for LRR-RLKs, representing a major class of receptors involved in microbe detection in plants through the recognition of MAMPs (54), suggesting that these genes are constitutively expressed in the red seaweed *L. dendroidea*.

Moreover, at 24 hpi, we verified the upregulation of the gene coding for NADPH oxidase, the major gene for ROS production in seaweeds, in *L. dendroidea* (14, 47, 55). Because ROS can react with essential host molecules, the activity of antioxidant enzymes is important to limit the oxidative burst. In this work, we report the upregulation of several antioxidant enzymes, especially TRX, PRX, GST, and SOD, 24 and 48 hpi. Accordingly, the expression of TRX, PRX, and GST increased in *Laminaria digitata* in response to oligoguluronates (19, 56) and the activity of SOD increased in *Saccharina japonica* as elicited with flg22, a MAMP (57).

### Activation of defense-related intracellular signaling cascades and transcription factors.

Signaling cascades that modulate the innate immune response have been well described in plants but are still unknown in seaweeds. Despite indirect evidence for the occurrence of mitogen-activated protein kinase (MAPK) cascades in seaweeds (49), the involvement of this pathway in the response to bacteria was not previously investigated. Here, we detected the upregulation in *L. dendroidea* of a gene coding for a MAPKK 24 hpi, indicating that a MAPK cascade was induced during the response of this seaweed to *V. madracius*. The MAPK cascade transduces extracellular stimuli into intracellular responses during plant defense against pathogens and can induce the expression of defense-related genes through the phosphorylation of transcription factors, such as ERF (58).

**FIG 4:**
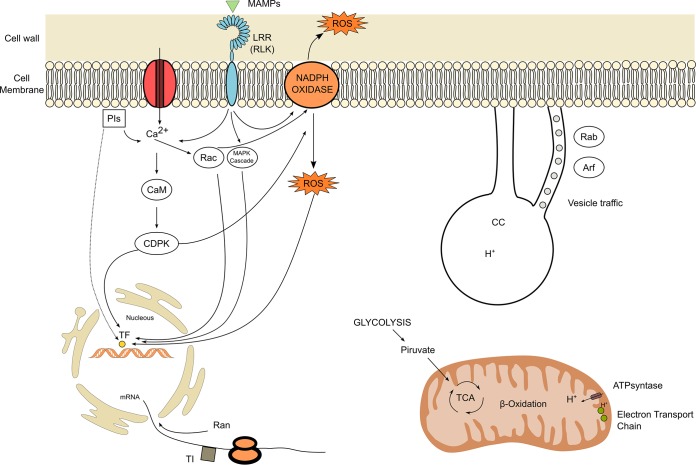
Hypothetical model representing bacterium recognition (through microbe-associated molecular pattern [MAMP]) and some relevant metabolic processes overrepresented in the transcriptomic profile of *Laurencia dendroidea* in response to *Vibrio madracius*. LRR (RLK), leucine-rich repeat receptor-like serine/threonine-protein kinase; ROS, reactive oxygen species; PIs, phosphatidylinositol signaling; Rac, Rho family GTPase Rac; CaM, calmodulin; CDPK, calcium calmodulin-dependent protein kinase; TF, transcription factors; Ran, nuclear protein Ran; TI, translation initiation factors; CC, *corps en cerise*; Arf, ADP-ribosylation factor; Rab, Rab GTPase; TCA, tricarboxylic acid. Note that the figure is not drawn to scale.

Further, we observed the upregulation of *L. dendroidea* genes coding for small GTPases, such as Rac, a member of the Rho family considered to be a key regulator in plant immunity, 24 hpi (59). The Rac1 homolog of rice is a regulator of ROS production and induces the expression of defense-related genes promoting resistance against pathogenic bacteria (60). Genes involved in PI signaling were also upregulated in *L. dendroidea* 24 hpi. Phosphoinositide-mediated signaling affects Ca^2+^ release and the expression of defense-related genes in plants (61). Indeed, we detected the upregulation of genes coding for CaM and CDPK 24 and 48 hpi which are required for sensing and decoding Ca^2+^ signals. Pathogenesis-related activation of CDPK was detected in plants (62), and this protein kinase regulates the production of ROS by NADPH ox (63). Another gene coding for a protein kinase upregulated in *L. dendroidea* 24 hpi was the Snf1-related protein kinase, whose expression in plants is induced by pathogenic bacteria (64). Further, a relevant role was attributed to Snf1-related protein kinases as global regulators of gene expression, inducing catabolic pathways that provide alternative sources of energy and controlling genes that encode signal transduction components and transcription regulators (65). Our work suggests that well-known mechanisms acting on the plant innate immunity response are also present in seaweeds (Fig. 4).

WRKY and MYB were upregulated in *L. dendroidea* 24 h after the inoculation of *V. madracius*, reinforcing the role of these transcriptional activators positively regulating genes related to immunity (66, 67). Similarly, both transcription factors were upregulated 12 h after peach leaves were inoculated with a pathogenic bacterial species (68) and MYB expression was induced 24 h after the inoculation of *Arabidopsis* with a pathogenic fungus (69). Another important transcriptional regulatory element upregulated in *L. dendroidea* 24 hpi was heat stress transcription factor (HSF), associated mainly with defense gene activation, pathogen-induced systemic acquired resistance (86), and transcriptional reprogramming in plants as a consequence of redirecting energy resources from growth to defense mechanisms (87).

Additionally, at 24 hpi, we detected the upregulation of an ERF that has diverse functions in plant defense and responds to jasmonic acid (JA) and ethylene (ET) (70, 71). There is evidence that JA, or a structurally similar compound(s), is also involved in defense signals in macroalgae, as this substance induced the expression of stress-related genes in *C. crispus* (22), increased the biosynthesis of phlorotannins in *Fucus vesiculosus* (72), and activated oxidative cascades in *Laminaria digitata* (47) and *C. crispus* (12). Although the role of ET signaling in seaweeds was not demonstrated, the ability to synthesize and respond to this plant hormone was previously detected in *Enteromorpha intestinalis* (73) and *Pterocladiella capillacea* (74). The present report contributes to evidence indicating the presence of a mechanism in seaweeds similar to plant hormone-regulated defense against microbes.

### Energy balance.

Diverse evidence suggests that fighting against microbes is energetically demanding in vascular plants (75). By using high-throughput transcriptome sequencing, we verified the transient upregulation, in response to *V. madracius*, of *L. dendroidea* genes involved in energy conversion, especially in relation to glycolysis, the tricarboxylic acid (TCA) cycle, and oxidative phosphorylation (Fig. 4). Further, we observed the upregulation of genes involved in the catabolism of branched-chain amino acids and in the β-oxidation of fatty acids, which provide alternative sources of respiratory substrates for the TCA cycle, especially during severe plant stress and in response to infection (76, 77).

### Secondary metabolites and defense.

The expression level of several genes involved in the biosynthesis of terpenes in *L. dendroidea* increased significantly 24 and 48 h after *V. madracius* inoculation. Terpenoid compounds are recognized as important secondary metabolites acting to defend *Laurencia* species against bacterial colonization (30). Indeed, acetyl-CoA C-acetyltransferase (overexpressed 24 and 48 hpi) catalyzes the first step in the biosynthesis of terpenoid backbones through the mevalonate pathway and was suggested to be a regulatory enzyme in isoprenoid biosynthesis during plant abiotic stress adaptation (78). Moreover, the upregulation of genes involved in monoterpene biosynthesis was detected in *L. dendroidea* in response to *V. madracius* and offers a possible explanation for the reduction in the concentration of these bacteria in the culture medium in the presence of the seaweed.

Genes relevant for vesicle trafficking—including those coding for Rab, which participates in intracellular membrane trafficking by regulating the movement of vesicles along cytoskeletal filaments (79); actin, which composes the structure of the connections linking the CC to the cell periphery in *L. dendroidea*; and tubulin, which is responsible for the positioning of the vesicles toward exocytosis sites—were upregulated in this seaweed in response to the inoculation of *V. madracius*, mainly 24 hpi (34). These findings may corroborate the occurrence of increased vesicle transport in *Laurencia* as a response to microbes (35).

The present report shows that even though *V. madracius* cannot be considered a pathogen of *L. dendroidea*, this seaweed is able to recognize and respond to the microbe through a temporal series of complex metabolic changes. Although we might expect the upregulation of genes related to microbe recognition, signaling, and oxidative burst to precede the upregulation of genes involved in terpene biosynthesis, future studies are needed to explore the timing of the expression of gene groups in shorter time periods (in the window of 24 h after bacterial inoculation). It is also necessary to determine if the measured differences represented a generic response of *Laurencia* to bacteria or a response to a specific potential pathogen.

### Conclusion.

The response of *L. dendroidea* to *V. madracius* involves transcriptomic reprogramming, especially 24 and 48 h after bacterial inoculation. The upregulation of genes coding for NADPH oxidase and antioxidant enzymes suggests the occurrence of an oxidative burst. Intracellular signaling mediated by a MAPK cascade, small GTPases, phosphatidylinositol, and calcium calmodulin-dependent protein kinases was observed as a seaweed response to bacteria. Further, the upregulation of genes related to the biosynthesis of terpenes, along with the overexpression of genes involved in vesicular transport, suggests increased release of terpenes by *L. dendroidea*. Finally, we verified the upregulation of genes associated with energy metabolism, indicating that the defense mechanisms in *L. dendroidea* might involve an energy cost. The upregulation of the genes involved in ROS production and in the biosynthesis of terpenes reveals a previously unknown integrated defensive system in seaweeds. The present study provided novel insights into the complexity of seaweed-microbe interactions and the defensive strategies of *L. dendroidea* at the molecular level.

## MATERIAL AND METHODS

*Laurencia dendroidea* (Hudson) J. V. Lamouroux was sampled at Castelhanos Beach in Anchieta municipality, Espírito Santo state (20°51′40″S, 40°37′00″W), and was maintained in a laboratory. The unialgal culture of this seaweed was established through successive excision of the apices. Clones were used to prevent intraspecific variations in transcriptomic profiles from masking the effect of bacterial inoculation. These algal clones were treated with 100 µg/ml ampicillin, 120 µg/ml streptomycin, and 60 µg/ml gentamicin, which reduced the levels of bacteria in the culture by more than 95%. The clones were grown in sterile seawater with germanium dioxide (1 mg/liter) and 50% Provasoli solution (enriched seawater medium [ESW]) for 2 days before the experiment. The culture and experimental conditions were as follows: temperature, 22 ± 1°C; salinity, 32 ± 1; irradiance, 80 ± 5 µmol photons ⋅ m^−2^ ⋅ s^−1^; 14 h light/10 h dark.

*Vibrio madracius* was isolated from the coral *Madracis decactis* sampled in Saint Peter and Saint Paul archipelago (40). The bacteria were grown at 30°C in sterile marine broth to an optical density at 600 nm (OD_600_) of 0.8, corresponding to 10^8^ CFU ⋅ ml^−1^, and precipitated for 5 min at 3,000 rpm (5415R centrifuge; Eppendorf). The supernatant was discarded, and the pellet was resuspended in sterile seawater and inoculated in Falcon tubes containing 250 mg of *L. dendroidea* and 40 ml of ESW (*n* = 2). The final concentration of *V. madracius* in the treatment was 10^7^ CFU ⋅ ml^−1^ (i.e., in the presence of *L. dendroidea*; replicates T1 and T2). The same quantity of bacteria was inoculated in Falcon tubes containing 40 ml of ESW (*n* = 2) in the absence of *L. dendroidea* (CV1 and CV2). The culture medium was plated in TCBS media (*n* = 3) immediately after bacterial inoculation and 24, 48, 72, 96, and 144 h after bacterial inoculation in the presence and absence of *L. dendroidea*. Also, 144 h after bacterial inoculation, the seaweed thalli were homogenized in a sterile 3% NaCl solution for 1 h using vortex mixing and this tissue homogenate was plated in TCBS media. The petri dishes were incubated overnight at 30°C, and the colonies of *V. madracius* were counted when present.

To evaluate the transcriptomic profile of *L. dendroidea* in the presence and absence of *V. madracius*, control tubes were set up with 250 mg of *L. dendroidea* and 40 ml of ESW (*n* = 3); the inoculated tubes contained 250 mg of *L. dendroidea*, 40 ml of ESW, and *V. madracius* at 10^7^ CFU ml^−1^ (*n* = 3). After 24, 48, and 72 h, control and inoculated *L. dendroidea* specimens were frozen and separately ground in liquid nitrogen using a mortar and pestle. Total RNA was extracted using the TRIzol (Life Technologies, Inc.) protocol. Double-strand cDNA libraries were prepared using a TruSeq stranded mRNA LT sample preparation kit (Illumina). Library size distribution was accessed using a model 2100 Bioanalyzer (Agilent) and a High Sensitivity DNA kit (Agilent). The accurate quantification of the libraries was accomplished using model 7500 real-time PCR (Applied Biosystems) and a Kapa library quantification kit (Kapa Biosystems). Paired-end sequencing (2 × 250 bp) was performed on a MiSeq sequencer (Illumina) for the following numbers of replicates: control 24 h = 2, inoculated 24 h = 3, control 48 h = 2, inoculated 48 h = 2, control 72 h = 3, inoculated 72 h = 3.

The sequences were preprocessed to trim poly(A-T) tails that were at least 20 bp long, to remove reads shorter than 35 bp, and to trim sequences with a quality score lower than Phred 30, using Prinseq software (80). The processed sequences from all of the samples were assembled using Trinity software, and sequences larger than 199 bp were used in the downstream analysis. Sequences from each sample were mapped against the assembled reads using Bowtie 2 (81) (with the following parameters: --end-to-end; --no-mixed; --no-discordant; --score-min L,-0.1,-0.1) and were clustered into genes using Corset software (minimum read count = 5) (82). A few bacterial sequences were detected through Blast searches against the NCBI-nr database and were removed from subsequent analysis. Statistically relevant genes differentially expressed between the control and the inoculated samples were identified using the edgeR software package associated with the Fisher exact test and Bonferroni correction for multiple tests, considering the following parameters: corrected *P* value, ≤0.001; log fold change [logFC] value, ≥2.0 (83). To plot a heat map of gene expression levels comparing control and inoculated samples (Fig. 2), we used *Z* score analysis, a conventional method of data normalization that calculates the mean expression value for a gene under the different conditions and normalizes the deviations as a function of the mean. The differentially expressed genes were annotated through a Blast search against the NCBI-nr database (E value, <10^−5^), and GO terms were assigned using the Blast2go tool (84). To identify the transcripts associated with the biosynthesis of terpenoid compounds, we analyzed the transcriptome of *L. dendroidea* using hidden Markov models generated from the alignment of sequences available in the KEGG database through the use of HMMER 3.0 software (85), following the method previously used by de Oliveira et al. (38). The sequences matching these profiles were annotated through a Blast search against the NCBI-nr, PlantCyc, and Uniprot databases. The functional identifications were manually confirmed.
