# Supplementary figures and images for: Molecular Mechanisms for Microbe Recognition and Defense by the Red Seaweed Laurencia dendroidea
Source: mSphere. 2017 Dec 6;2(6):e00094-17. doi: 10.1128/mSphere.00094-17 (PMC5717322; doi:10.1128/mSphere.00094-17)

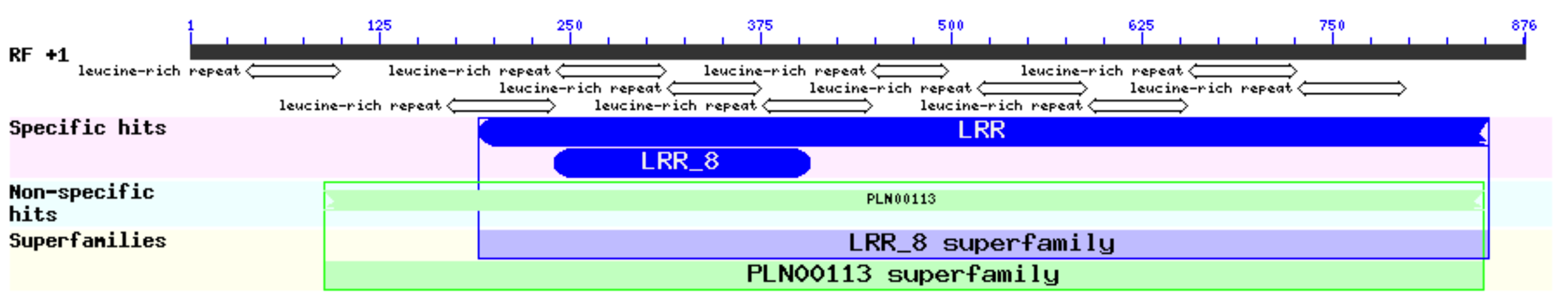

Supplement: FIG S2 [file sph006172418sf2.tif]

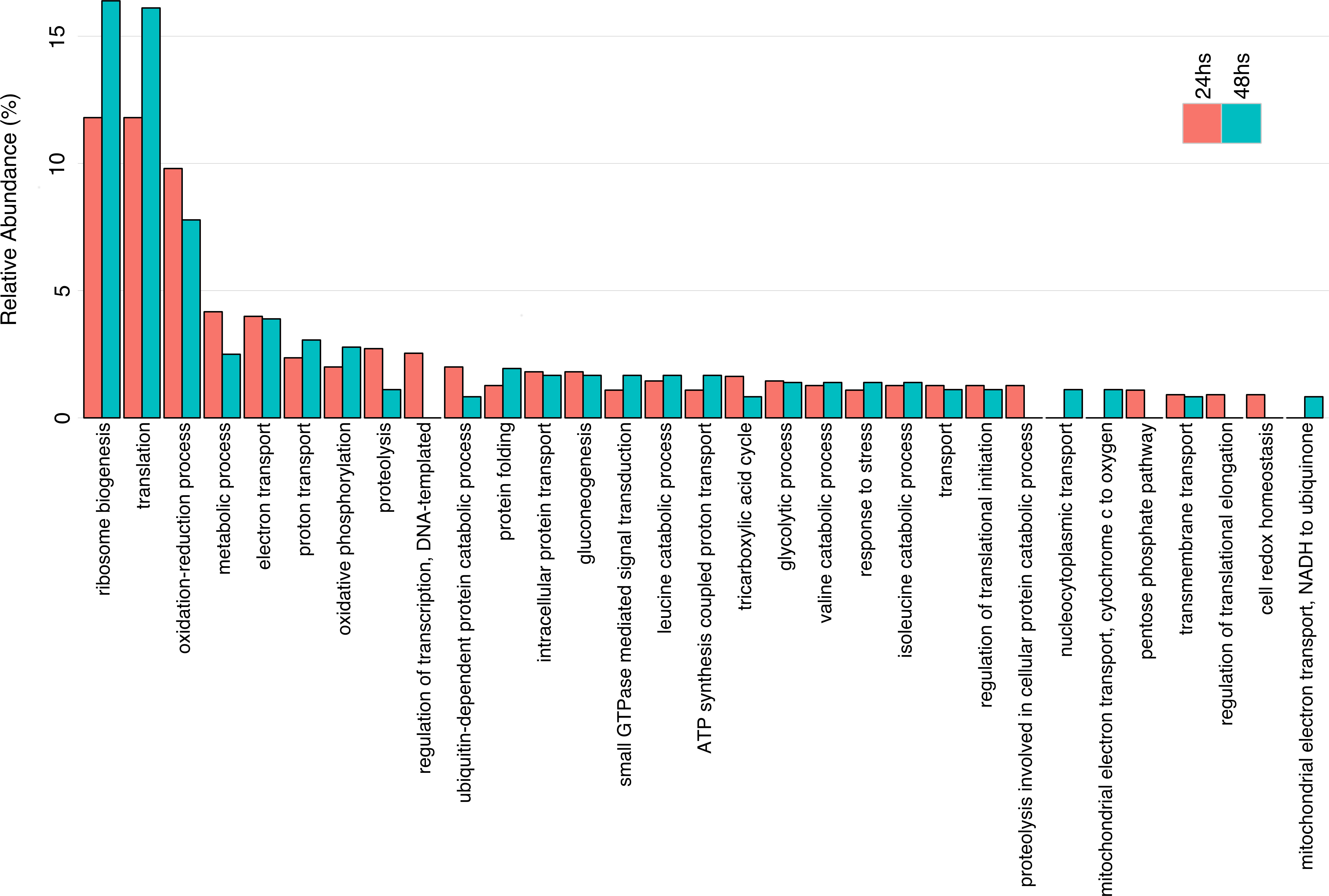

Supplement: FIG S3 [file sph006172418sf3.jpg]
